# Incidence of total hip or knee replacement due to osteoarthritis in relation to thyroid function: a prospective cohort study (The Nord-Trøndelag Health Study)

**DOI:** 10.1186/s12891-017-1565-6

**Published:** 2017-05-18

**Authors:** Alf Inge Hellevik, Marianne Bakke Johnsen, Arnulf Langhammer, Anne Marie Fenstad, Ove Furnes, Kjersti Storheim, John Anker Zwart, Gunnar Flugsrud, Lars Nordsletten

**Affiliations:** 1 The HUNT Research Centre, Department of Public Health and Nursing, Faculty of Medicine and Health Sciences, Norwegian University of Science and Technology (NTNU), Levanger, Norway; 2 Division of Orthopaedic Surgery, Oslo University Hospital, Oslo, Norway; 3 Research and Communication Unit for Musculoskeletal Health, Division of Clinical Neuroscience, Oslo University Hospital, Oslo, Norway; 4 The Norwegian Arthroplasty Register, Department of Orthopedic Surgery, Haukeland University Hospital, Bergen, Norway; 5 Department of Clinical Medicine, Institute of Medicine and Dentistry, University of Bergen, Bergen, Norway; 6 Faculty of Medicine, University of Oslo, Oslo, Norway

**Keywords:** Thyroid function, Thyroid stimulating hormone, Osteoarthritis, Hip joint replacement, Knee joint replacement

## Abstract

**Background:**

To study whether thyroid function was associated with risk of hip or knee replacement due to primary osteoarthritis.

**Methods:**

In a prospective cohort study, data from the second and third survey of the Nord-Trøndelag Health Study were linked to the Norwegian Arthroplasty Register in order to identify total hip or knee replacement as a result of primary osteoarthritis.

**Results:**

Among 37 891 participants without previously known thyroid disease we recorded 978 total hip replacements (THRs) and 538 total knee replacements (TKRs) during a median follow-up time of 15.7 years. The analyses were adjusted for sex, age, BMI (body mass index), smoking, physical activity and diabetes. We did not find any association between TSH (thyroid stimulating hormone) and THR or TKR due to osteoarthritis. Neither were changes in TSH over time, or overt hypo- or hyperthyroidism, associated with incidence of THR or TKR.

**Conclusion:**

No association was found between thyroid function and hip or knee joint replacement due to osteoarthritis.

## Background

Osteoarthritis in the hip and knee is a major health problem and leads to significant morbidity [1]. Several drugs have been evaluated, but so far only exercise has been found to effectively prevent or delay the onset of osteoarthritis [2, 3]. This finding emphasizes the importance of identifying modifiable risk factors. Thyroid hormones play a role in the remodelling and maintenance of bone, and recent studies also indicate the potential importance of thyroid hormones in joints and articular cartilage [4]. Genetic studies have suggested that deiodinase-regulated local availability of the active thyroid hormone triiodothyronine (T3) plays an important role in cartilage maintenance and repair [5]. Further data have indicated that increased intracellular T3 availability increases the risk of osteoarthritis, leading to the hypothesis that reduced tissue T3 availability protects joints from development of osteoarthritis [6]. A phase III clinical trial investigating the use of eprotirome, a thyroid receptor β-agonist, for treatment of hypercholesterolemia [7], was terminated due to indications of dose related articular cartilage damage in dogs which had been treated with eprotirome for 12 months [8]. This was surprising, as eprotirome is a liver-specific thyroid receptor β-agonist, but it indicates that thyroid hormones influence cartilage [9] and could play a role in the pathogenesis of osteoarthritis.

No prospective population studies have investigated the association between thyroid function and osteoarthritis. An older cross-sectional study did not find any association between radiological knee osteoarthritis and thyroid status measured by thyroid stimulating hormone (TSH) [10]. In this prospective cohort study of 37 891 individuals without previously known thyroid disease, the aim was to assess whether thyroid function was associated with subsequent risk of hip or knee replacement due to primary osteoarthritis.

## Methods

In the Nord-Trøndelag Health Study (HUNT) all inhabitants of Nord-Trøndelag county ≥ 20 years of age were invited to participate in three surveys: HUNT1 (1984–1986), HUNT2 (1995–1997) and HUNT3 (2006–2008) [11]. This study only included data from the HUNT2 and HUNT3 surveys, as the HUNT1 study did not collect blood samples. HUNT2 had 65 237 participants (69.5% of those invited), and HUNT3 had 50 807 participants (54.1% of those invited) [12].

**Fig. 1:**
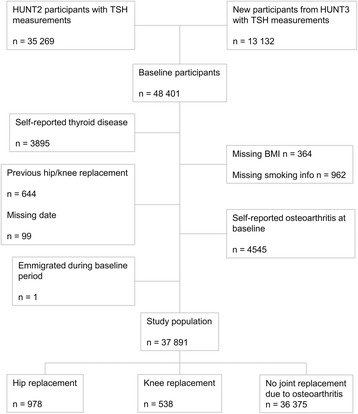
Flowchart (TSH: Thyroid stimulating hormone)

In HUNT2, TSH was measured in 35 269 persons; in all women over 40 years old, in a random 50% sample of men over 40 years old and in a random 5% sample of participants aged 20–40 years. In HUNT3, TSH was measured in all 49 179 participants. We included 35 269 participants from HUNT2 and 13 132 new participants with TSH measurements from HUNT3. In persons that participated in both HUNT2 and HUNT3, baseline measurements from HUNT2 were used in the main analyses. Among these 48 401 individuals, 10 510 were excluded from analysis (Fig. 1). The exclusion criteria included self-reported thyroid disease (hypothyroidism, hyperthyroidism, goitre, other thyroid disease, use of levothyroxine, carbimazole, previous thyroid surgery or radioiodine therapy) (*n* = 3895), missing information on BMI (*n* = 364), missing information on smoking (*n* = 962), previous THR or TKR (*n* = 644), missing date of operation (*n* = 99), emigration during baseline measurements period (*n* = 1) or self-reported osteoarthritis at baseline (*n* = 4545). Thus, a total of 37 891 people (22 714 women and 15 177 men) were eligible for follow-up in this study. Each participant contributed person-years from baseline (either between August 1995 and June 1997 or between October 2006 and June 2008) until a THR or TKR due to osteoarthritis, THR or TKR due to other causes, migration, death or end of follow-up (December 31, 2013), whichever occurred first.

### Measurements

The participants filled out a self-administered questionnaire, including history of thyroid disease [13]. The survey also included measurements of height and weight by trained personnel. Weight was measured while the participants were wearing light clothing without shoes. BMI was calculated as weight in kilograms divided by squared height in metres.

A non-fasting venous blood sample was drawn from each participant. Concentrations of TSH, free thyroxine (fT4) and total triiodothyronine (T3) in HUNT2 were measured at the Hormone Laboratory, Aker University Hospital, Oslo, using DELFIA hTSH Ultra (sensitivity, 0.03 mU/L; and total analytic variation <5%), DELFIA fT4 (total analytic variation <7%), and AutoDELFIA T3 (total analytic variation <5%), all from Wallac Oy, Turku, Finland. In HUNT3, serum TSH and fT4 were measured at Levanger Hospital, Levanger, Norway, using Architect cSystems ci8200 (sensitivity, 0.01 mU/l; and a total analytic variation <5%), and Architect cSystems ci8200 (total analytic variation <6%), respectively, both from Abbott, Clinical Chemistry, USA. The measurement methods of TSH in HUNT2 and HUNT3 have previously been compared, with similar results [14], and agreement expressed by Bland-Altman [15] did not reveal any obvious pattern or deviations. The Norwegian population is considered to have sufficient iodine intake [16], and reference range for clinically normal TSH was defined as 0.50 to 3.5 mU/l based on previous publications from this population [13].

### Covariates

The covariates were chosen based on previous literature, and had to be associated with both thyroid function and osteoarthritis. We mapped possible casual pathways between all variables using a directed acyclic graph model (DAG) (Appendix) to visually identify possible confounding pathways [17]. Based on this DAG-model, the possible confounders included in this analysis were sex, age (continuous), BMI (continuous), current smoking status (never, former or current), diabetes (yes or no) and physical activity. Physical activity was categorized by duration of light physical activity (none, <1, 1–2, ≥3 h/week), and/or duration of hard physical activity (none, <1, 1–2, ≥3 h/week). The physical activity questions have been previously validated among men, where especially hard physical activity correlated well with more objective measures [18]. These two variables were combined into one physical activity variable indicating intensity and duration: None (no activity), medium (≤2 h/week light physical activity and/or < 1 h/week hard physical activity), hard (≥ 3 h/week light physical activity and/or ≥ 1 h/week hard physical activity).

Osteoarthritis at baseline was based on an affirmative answer to the question: “Has a doctor ever said that you have/have had osteoarthritis?” from HUNT2 participants, and “Have you had or do you have osteoarthritis?” from HUNT3 participants.

### Outcome

In this study, primary total hip or knee replacement was considered to be a surrogate measure of severe osteoarthritis; and we used primary total hip or knee replacement due to primary osteoarthritis as outcome. The 11-digit identification numbers assigned to every Norwegian citizen enabled linkage to the Norwegian Arthroplasty Register (NAR). For each arthroplasty performed, the orthopaedic surgeon submits a standardized form containing information about the patient, the diagnosis that led to the arthroplasty, the procedure and the type of implant used [19]. NAR was established in 1987 and includes all artificial joints from 1994 onwards. The completeness of hip and knee replacement registration is over 95% [20].

### Statistical methods

The participants were placed in five categories according to their TSH level: One category indicating hyperthyroid function (<0.50 mU/l); three categories within the clinical reference range (0.50–1.49, 1.5–2.49 and 2.5–3.5 mU/l); and one category indicating hypothyroid function (≥3.5 mU/l) [21]. Hazard ratios (HRs) of THR or TKR by category of TSH were estimated using a Cox proportional hazards model with 95% confidence interval (CI); TSH 1.5–2.49 mU/l was chosen as a reference. TSH was also analysed as both a continuous variable, and as log-transformed continuous TSH. The HRs were adjusted for age, sex, BMI and smoking. An additional analysis also adjusted for physical activity and diabetes. We treated THR and TKR both as separate outcomes and combined in one outcome variable of total joint replacement (TJR).

A sub-analysis included only persons that participated in both HUNT2 and HUNT3 (*n* = 19 397). TSH levels at HUNT3 were then subtracted from the TSH levels in HUNT2 to estimate the change in TSH during the intervening decade. Information on the other baseline covariates was taken from HUNT3. Among these participants a total of 7740 were excluded due to self-reported thyroid disease (*n* = 2744), missing information on BMI (*n* = 108), missing information on smoking (*n* = 621), previous THR or TKR (*n* = 928), missing date of operation (*n* = 29) or self-reported osteoarthritis at baseline (*n* = 3310). Thus, a total of 11 657 people were eligible for this sub-analysis. This sub-population was also used in an analysis that investigated whether the results changed when those who had started on thyroid medication after baseline in HUNT2 were excluded.

In a sensitivity analysis people were divided into two groups, one with biochemically manifest (overt) hypothyroidism (defined as TSH >4.0 mU/L combined with fT4 < 8.0 pmol/L), and the other with overt hyperthyroidism (defined as TSH <0.10 mU/L and fT4 > 20.0 pmol/L and/or total T3 > 2.7 nmol/L). This classification by overt hypo- or hyperthyroidism was made possible by the fT4 measurements taken in people whose TSH levels were <0.20 mU/l or >4.0 mU/l in HUNT2, and in people whose levels were <0.10 mU/l or >3.0 mU/l in HUNT3. Total T3 was only available in HUNT2 and only measured if TSH levels were <0.20mU/L.

Two additional analyses were performed on the baseline population: First, we investigated the association between TSH level (categorical) and self-reported osteoarthritis at baseline by using a logistic regression model, adjusting for sex, age, BMI, smoking, physical activity and diabetes. Second, we compared the incidence rate of THR or TKR in participants with and without self-reported thyroid disease at baseline using Fisher’s Exact test. After excluding participants with missing information on BMI and smoking, previous THR or TKR, missing date of operation or self-reported osteoarthritis at baseline, 2955 participants reported thyroid disease.

Proportional hazards assumptions were evaluated by Schoenfeld residuals tests. They showed proportional hazards on all covariates, except for age. Thus we did an additional stratified analysis on age, but it did not show different results (data not shown). Age was therefore kept as a continuous variable in all our analyses. All statistical analyses were two-sided with a significance level of *p* < 0.05. The analyses were performed using Stata 14.0/SE (StataCorp LP, College Station, TX, USA).

## Results

Of the 37 891 participants, 908 (2.4%) had low TSH (<0.50 mU/l) indicating hyperthyroid function, and 2307 (6.1%) had high TSH (≥3.6 mU/l) indicating hypothyroid function (Table 1). Among the women, 6.9% had high TSH, compared to 4.8% of men. Participants with high TSH were generally older and were less likely to be current smokers than participants in the reference group (TSH 1.5–2.4 mU/l). No clear trend was seen in relation to physical activity.

**Table 1:** Study population characteristics in relation to thyroid stimulating hormone (TSH) categories

			Serum TSH								Total	
	*<0.50*		*0.50–1.49*		*1.5–2.49*		*2.5–3.49*		*≥3.5*			
Participants (%)	908	(2.4)	17675	(46.7)	13228	(34.9)	3773	(10.0)	2307	(6.1)	37891	
Women (%)	651	(2.9)	10405	(45.8)	7738	(34.1)	2346	(10.3)	1574	(6.9)	22714	(60.0)
Men (%)	257	(1.7)	7270	(47.9)	5490	(36.2)	1427	(9.4)	733	(4.8)	15177	(40.0)
Age (SD)	50.7	(16.7)	48.2	(15.3)	51.6	(15.6)	54.9	(15.9)	58.3	(15.3)	50.7	(15.8)
BMI (SD)	25.9	(4.4)	26.1	(4.1)	26.8	(4.2)	27.1	(4.4)	27.2	(4.5)	26.5	(4.2)
Smoking status (%)												
Never	335	(36.9)	6778	(38.3)	6080	(46.0)	1869	(49.5)	1167	(50.6)	16229	(42.8)
Former	236	(26.0)	4683	(26.5)	3768	(28.5)	1120	(29.7)	723	(31.3)	10530	(27.8)
Current	337	(37.1)	6214	(35.2)	3380	(25.5)	784	(20.8)	417	(18.1)	11132	(29.4)
Physical activity (%) (missing = 6826)												
Low	46	(6.5)	862	(6.0)	719	(6.6)	232	(7.4)	151	(8.2)	2010	(6.5)
Medium	371	(52.8)	7226	(49.9)	5454	(50.0)	1555	(49.7)	921	(50.1)	15527	(50.0)
High	286	(40.7)	6394	(44.1)	4738	(43.4)	1344	(42.9)	766	(41.7)	13528	(43.6)
Diabetes (%) (missing = 56)	55	(6.1)	613	(3.5)	559	(4.2)	173	(4.6)	115	(5.0)	1515	4.0

In total, 978 received THR and 538 received TKR during a median follow-up time of 15.7 years (mean 12.3 years). At baseline, the mean age was 50.7 years (SD 15.8), and the mean ages at THR and TKR were 69.5 years (SD 8.9) and 69.4 years (SD 8.4), respectively.

**Fig. 2:**
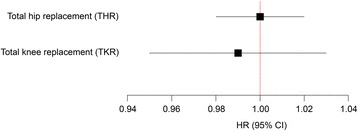
Association between continuous TSH and total hip replacement (THR) or total knee replacement (TKR) due to osteoarthritis. (Adjusted for age, sex, BMI, smoking, physical activity and diabetes)

TSH level did not influence the risk of THR or TKR in the unadjusted analysis or the analysis adjusted for gender, age, BMI and smoking (Table 2). Neither additional adjustment for physical activity and diabetes (Table 2), nor collapsing the outcome variable into total joint replacement (TJR) (Table 3), altered these results. Analyses using TSH as a continuous variable did not show any association between TSH and THR or TKR (Fig. 2). Additional log-transformation of TSH as a continuous variable did not significantly change these results (data not shown).

**Table 2:** Association between TSH categories and hip or knee replacement due to osteoarthritis

TSH (mU/L)	Persons (n)	Cases (n)	HR^a^	(95% CI)	HR^b^	(95% CI)	HR^c^	(95% CI)
THR								
< 0.50	908	21	0.98	(0.63–1.53)	0.99	(0.64–1.53)	1.04	(0.63–1.69)
0.50–1.49	17675	411	0.93	(0.81–1.08)	1.07	(0.93–1.24)	1.09	(0.94–1.28)
1.5–2.49	13228	359	1	Reference	1	Reference	1	Reference
2.5–3.49	3773	105	0.98	(0.79–1.22)	0.85	(0.68–1.05)	0.88	(0.69–1.11)
> 3.5	2307	82	1.22	(0.96–1.55)	0.96	(0.76–1.22)	0.98	(0.75–1.27)
TKR								
< 0.50	908	14	1.26	(0.73–2.16)	1.32	(0.76–2.26)	1.33	(0.74–2.4)
0.50–1.49	17675	224	0.97	(0.80–1.18)	1.15	(0.95–1.40)	1.12	(0.91–1.37)
1.5–2.49	13228	191	1	Reference	1	Reference	1	Reference
2.5–3.49	3773	64	1.12	(0.84–1.48)	0.94	(0.71–1.25)	0.88	(0.65–1.20)
> 3.5	2307	45	1.24	(0.90–1.72)	0.98	(0.70–1.35)	0.87	(0.61–1.25)

(*THR* total hip replacement, *TKR* total knee replacement)

^a^Unadjusted

^b^Adjusted for age, sex, BMI and smoking

^c^Adjusted for age, sex, BMI, smoking, physical activity and diabetes

**Table 3:** Association between TSH categories and total joint replacement (TJR) in the hip or knee due to osteoarthritis

TSH (mU/L)	Persons (n)	Cases (n)	HR^a^	(95% CI)	HR^b^	(95% CI)	HR^c^	(95% CI)
<0.50	908	35	1.08	(0.77–1.52)	1.10	(0.78–1.55)	1.15	(0.79–1.67)
0.50–1.49	17675	635	0.95	(0.84–1.06)	1.10	(0.98–1.23)	1.10	(0.97–1.25)
1.5–2.49	13228	550	1	Reference	1	Reference	1	Reference
2.5–3.49	3773	169	1.03	(0.87–1.22)	0.88	(0.74–1.05)	0.88	(0.73–1.06)
>3.5	2307	127	1.23	(1.01–1.49)	0.97	(0.80–1.17)	0.94	(0.76–1.16)

^a^Unadjusted

^b^Adjusted for age, sex, BMI and smoking

^c^Adjusted for age, sex, BMI, smoking, physical activity and diabetes

**Fig. 3:**
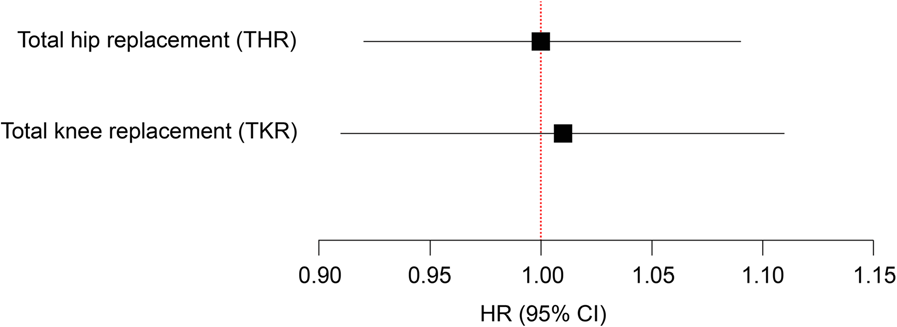
Association between change in TSH (delta TSH) and total hip replacement (THR) or total knee replacement (TKR) due to osteoarthritis. (Adjusted for age, sex, BMI and smoking)

In a separate analysis studying the change in TSH (delta-TSH) during the time between HUNT2 and HUNT3, 11 657 participants were included. Of these, 200 received THR and 102 received TKR during a mean follow-up time of 6.1 years. No association was seen between changes in TSH and risk of THR or TKR (Fig. 3). Additional adjustment for baseline TSH did not substantially alter these results. The same sub-population was also used to identify persons with no thyroid disease or treatment at baseline in HUNT2 who still had no thyroid disease or treatment at HUNT3. However, no association between TSH and THR (HR 1.00, 95% CI 0.97–1.02) or TKR (HR 0.98, 95% CI 0.94–1.03) was found.

In a sensitivity analysis neither overt hypo- nor hyperthyroid function was found to influence the risk of THR or TKR, (HR 0.91, 95% CI 0.40–2.04) and (HR 2.01, 95% CI 0.75–5.4) respectively. Also, no association was found between overt hypothyroid function and TKR (HR 1.03, 95% CI 0.38–2.78). There were no cases of TKR amongst the overtly hyperthyroid, so no analysis could be performed in this subgroup.

In additional analyses on the baseline population, there was no association between TSH levels and self-reported osteoarthritis at baseline (data not shown). Those with self-reported thyroid disease at baseline had an incidence rate of 0.0027 THR per person-year and 0.0015 TKR per person-year. Our main study population (excluding those with self-reported thyroid disease) had an incidence rate of 0.0021 THR per person-year and 0.0012 TKR per person-year. This gave and incidence rate ratio of 1.28 (95% CI 1.04–1.56) for THR, and 1.27 (95% CI 0.95–1.95) for TKR, and indicated a slightly higher incidence rate for THR in those with self-reported thyroid disease, but no significant difference in the incidence rate for TKR, compared to participants without self-reported thyroid disease.

## Discussion

In this large prospective study we did not find any association between thyroid function and the risk of THR or TKR due to osteoarthritis. Neither were changes in TSH over time, or overt hypo- or hyperthyroidism, associated with incidence of THR or TKR.

Few previous population studies have investigated the association of thyroid function with risk of osteoarthritis. In 1996, a cross-sectional study of 577 men and 798 women found no evidence of a significant association between current thyroid status and either chondrocalcinosis or osteoarthritis [10]. However, that study only investigated prevalent osteoarthritis with a concurrent serum TSH concentration and could not take into account development in TSH or later treatment for abnormal thyroid function. Since our study could use data from both the second and third waves of the HUNT-survey, we were able to investigate the development of TSH over a median time of 11.2 years (SD 0.6). Change in TSH over time was however not associated with osteoarthritis development resulting in the need for joint replacement.

Previous or current thyroid disease at baseline was an exclusion criterion in our study. Nonetheless, all participants with TSH levels suggesting hypothyroid or hyperthyroid function may have received medical treatment for thyroid disease during the follow-up period, as participants with biochemical indication of pathological thyroid function were recommended to contact their general practitioners [22]. This could have weakened any association between TSH and osteoarthritis. We therefore did a sub-analysis of persons that participated in both HUNT2 and HUNT3, and excluded participants that reported use of thyroid medication or thyroid disease in HUNT3. This did not significantly alter the results.

Our findings must be interpreted in relation to recent genetic studies on intracellular T3 availability in joint cartilage. There has been an increased interest in the effect of deiodinase polymorphisms on osteoarthritis [5]. Iodothyronine deiodinases represent a family of proteins involved in local homeostasis of thyroxine (T4) and triiodothyronine (T3). Three deiodinases have been described and, of these, the deiodinase type 2 (D2) and deiodinase type 3 (D3) are detected in bone and cartilage. D2 plays a major role in conversion of T4 to biologically active T3 [23] and thus upregulates local T3 levels. Deiodinase type 3 (D3) is the main T3-inactivating enzyme and consequently downregulates the local T3 levels. T3 is considered an important regulator of chondrocyte cell growth and differentiation in the endochondral growth plate [24]. Local T3 availability, regulated by the opposite functions of D2 and D3, may be a determinant of osteoarthritis development. D2 has been reported to be upregulated in the cartilage of joints affected by osteoarthritis compared to joints unaffected by osteoarthritis [25, 26]. However, it is not known if this is a result of the ongoing osteoarthritis process, or a reflection of the underlying disease pathway. Taken together, these findings suggest that deiodinase regulated local availability of T3 in chondrocytes is a possible factor in the pathophysiology of osteoarthritis [27, 28]. Since our study did not find any association between circulating TSH, T3/T4 levels and osteoarthritis, it is conceivable that the serum thyroid hormone levels may be independent of local intracellular T3 levels in joints. Another possible explanation could be that polymorphism in the gene coding for D2 creates a predisposition for non-optimal bone shape [29, 30], leading to increased risk of osteoarthritis independent of local thyroid hormone levels.

### Strengths and limitations

Our study included over 37 000 persons without known thyroid disease at baseline, and in most cases thyroid function was measured many years prior to joint replacement. To the best of our knowledge, this is the first prospective population study addressing the association between thyroid function and joint replacement due to primary osteoarthritis. The prospective design and longitudinal data on TSH measurements are strengths of this study. By excluding participants with self-reported osteoarthritis at baseline it was possible to differentiate between risk of osteoarthritis development and progression.

Our study used joint replacement due to primary osteoarthritis as a surrogate measure of severe osteoarthritis. Validation of the osteoarthrosis diagnosis from the Norwegian Arthroplasty Register has not been done in an unselected population [31]. However, the Danish hip Arthroplasty Registry has reported a positive predictive value of 85% regarding primary hip osteoarthritis diagnosis [32], and it is likely that these results are comparable to the Norwegian Arthroplasty Register. The advantage of using joint replacement as a proxy for osteoarthritis is its unambiguous connection with disease burden of osteoarthritis compared to other osteoarthritis definitions, e.g., radiographic criteria, symptom criteria or osteoarthritis defined by self-reported diagnosis [33]. However, this outcome measure is still limited in some respects, most importantly, that patients’ health status and potential comorbidities influence orthopaedic surgeons’ choices regarding operative treatment. Secondly, persons with moderate osteoarthritis who engage in demanding physical activities could be more motivated to have surgery than less active persons. Persons who are generally inactive may be less motivated to have surgery even if they have more severe osteoarthritis. This could give a healthy patient selection bias with corresponding underestimation of the effect of thyroid function.

Previous injuries increase the risk of knee osteoarthritis [34, 35], but only joint replacements due to primary/idiopathic osteoarthritis were included in our study. We did not have direct information on previous injury, but we excluded all cases in which the operating surgeon reported that the knee joint replacement was due to sequela from fracture, ligament injury, meniscal injury, infection, rheumatoid arthritis or ankylosing spondylitis.

The interrelationship between BMI and thyroid function is complex and BMI could be treated as either a confounder or a mediator in our model [36]. The reason we chose to define it as a confounder was that we wanted to investigate the direct effect of thyroid function on joint replacement, independent of BMI. Potential confounding by other unmeasured factors could not be excluded. But these factors should then be associated with both thyrotropin level and osteoarthritis. Therefore, we did not adjust for level of education in this study (Appendix).

The participation rate in the HUNT-surveys was fairly high compared to most other surveys, but there is always a potential risk of selection bias that cannot be adjusted for in the statistical analysis [37]. Blood samples were not drawn at a set time of the day, and it is known that other factors like exercise and sleep deprivation influence TSH levels [38]. This might have led to non-differential classification bias, thus weakening any associations. We also only had data on fT4 and T3 in subpopulations. Therefore, the absolute numbers of participants with overt hypo- or hyperthyroidism were small, reducing the power to detect any association between them and euthyroid subject and should thus be interpreted with caution.

This study focused on the relationship between thyroid function and osteoarthritis. However, there may also be an association between autoimmune thyroid disease and osteoarthritis [39]. As thyroid autoantibody not necessarily correlate with thyroid function, an association between thyroid function and osteoarthritis trough autoimmune factors could be missed in our study.

The exclusion of participants who reported hypo- or hyperthyroidism in their answers regarding use of treatment or medication might have caused misclassification since radioiodine is used in the treatment of cancer and T4 in the medical treatment of goitre. However, these treatments are infrequent, and it is unlikely that they substantially altered our results.

In the analysis comparing incidence rate of THR in people with and without self-reported thyroid disease we found a small, but significant, increased risk of THR in people reporting thyroid disease. And since we excluded participants with self-reported thyroid disease, this might have led to an underestimation of the effect on THR. We therefore did an additional analysis including those with self-reported thyroid disease: This showed no association between TSH levels and THR in a Cox-regression model (data not shown), and thus confirmed the findings from the primary analysis.

## Conclusion

In this prospective study of 37 891 participants without previously known thyroid disease, we did not find that thyroid function was associated with risk of hip or knee joint replacement due to osteoarthritis. Neither did we find any association between TSH development over time and risk of hip or knee joint replacement due to osteoarthritis.

## Figures and Tables

**Fig. 4:**
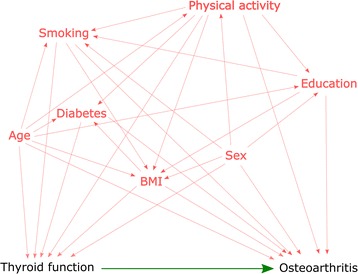
Direct Acyclic Graph (DAG) – analysis. Figure showing possible casual pathways between variables

## Abbreviations

BMI: Body mass index
D2: Deiodinase type 2
D3: Deiodinase type 3
DAG: Directed acyclic graph
fT4: Free thyroxine
HR: Hazard ratio
HUNT: The Nord-Trøndelag Health study
NAR: Norwegian Arthroplasty Register
OA: Osteoarthritis
SD: Standard deviation
T3: Triiodothyronine
T4: Thyroxine
THR: Total hip replacement
TKR: Total knee replacement
TSH: Thyroid stimulating hormone
